# Gene expression identifies patients who develop inflammatory arthritis in a clinically suspect arthralgia cohort

**DOI:** 10.1186/s13075-020-02361-2

**Published:** 2020-11-09

**Authors:** Ellis Niemantsverdriet, Erik B. van den Akker, Debbie M. Boeters, Susan J. F. van den Eeden, Annemieke Geluk, Annette H. M. van der Helm-van Mil

**Affiliations:** 1 Department of Rheumatology, Leiden University Medical Center, PO Box 9600, Leiden, 2300 RC The Netherlands; 2 Department of Biomedical Data Sciences, Leiden University Medical Center, Leiden, The Netherlands; 3 Pattern Recognition & Bioinformatics, Delft University of Technology, Delft, The Netherlands; 4 Department of Infectious Diseases/Immunohematology and Blood Transfusion, Leiden University Medical Center, Leiden, The Netherlands; 5 Department of Rheumatology, Erasmus Medical Center, Rotterdam, The Netherlands

**Keywords:** Clinically suspect arthralgia, RNA, Gene expression, MLPA, Inflammatory arthritis

## Abstract

**Background:**

Established rheumatoid arthritis (RA) patients display differentially expressed genes coding for cytokine/chemokine-mediated immunity compared to healthy controls. It is unclear, however, if in the pre-arthritis phase of clinically suspect arthralgia (CSA) expression of immune genes differ between patients who do or do not develop clinically evident inflammatory arthritis (IA).

**Methods:**

Two hundred thirty-six consecutive patients presenting with arthralgia clinically suspected for progression to RA were followed until IA development or else for median 24 months (IQR 12–26). Baseline whole blood RNA expression was determined for a previously identified set of 133 genes associated with the innate and adaptive immune system by dual-color reverse-transcription multiplex ligation-dependent probe amplification (dcRT-MLPA) profiling. Cox proportional hazard models were used.

**Results:**

Twenty percent of CSA patients developed IA. After correction for multiple testing, expression levels of six genes (IFNG, PHEX, IGF-1, IL-7R, CD19, CCR7) at the time of presentation were associated with progression to IA. PHEX and IGF-1 were highly correlated with each other (*ρ* = 0.97). In multivariable analysis correcting for the different genes, expressions of IL-7R and IGF-1 were independently associated with IA development (*p* = 0.025, *p* = 0.046, respectively). Moreover, IL-7R and IGF-1 remained significantly associated even after correction for known predictors (ACPA, CRP, imaging-detected subclinical joint inflammation; *p* = 0.039, *p* = 0.005, respectively). These genes are also associated with RA development.

**Conclusions:**

IL-7R and IGF-1 were differentially expressed between CSA patients who did or did not progress to IA, independent from regularly used predictors. These biomarkers may become helpful in prognostication of CSA patients. Furthermore, because both genes are associated with T cell functioning, T cell dysregulation may mediate progression from arthralgia to arthritis.

## Background

Rheumatoid arthritis (RA) is characterized by chronic inflammation, mostly of small joints. Although clinical arthritis is mandatory for diagnosing, the large majority of RA patients have joint symptoms for a period of time before clinical arthritis (joints swelling) develops. During development of RA, the occurrence of clinical arthritis is preceded by a phase where patients have symptoms, which are recognized by rheumatologists as arthralgia suspicious for progression to RA (clinically suspect arthralgia, CSA). However, only a proportion of patients that present with CSA actually progress to clinical arthritis and RA. Identified predictors mainly fall into three categories: autoantibodies, inflammatory markers measured in the systemic circulation, and imaging detected subclinical joint inflammation [1]. Most CSA patients do not show all these risk factors and current prediction making is insufficiently accurate. The identification of additional biomarkers is therefore required. Furthermore, the pathophysiology of RA development is still incompletely understood, and it is unclear which processes are related to the final hits that mediate progression from arthralgia to clinical arthritis and RA.

Although autoantibodies and imaging-detected inflammation in arthralgia are extensively explored as biomarkers [1], the value of inflammatory response proteins measured in the systemic circulation is less clear. Most research has so far been conducted on C-reactive protein (CRP), which is routinely measured in the clinic, but its predictive value in arthralgia is not undisputed [2]. Furthermore, CRP has little relationship with the wide variety of inflammatory and immune response proteins that are measurable in the systemic circulation. Nested case-control studies have shown that proinflammatory cytokines can be increased months prior to the diagnosis of RA [3], though nested case-control studies have the disadvantage that controls are selected and that prospective data from non-progressing patients in a similar pre-disease stage are absent. Longitudinal cohort studies in at-risk cohorts are therefore needed. A few gene expression studies have been performed in cohorts with autoantibody-positive arthralgia patients. These showed differentially expressed genes coding for cytokine/chemokine-mediated (among interferon gamma (IFNG), interleukin-7 receptor (IL-7R)) and interferon-mediated immunity in patients that progressed to RA [4, 5]. Also, a B cell signature and T cell subset dysregulation have been described [6, 7]. Together, these data support the hypothesis that genes related to immunity and inflammation in the systemic circulation are differently produced between patients with CSA that progress to RA versus those who do not progress.

As previous reports suggest the involvement of immune response genes in RA pathology [4–7], we here hypothesize that the expression of genes related to immunity and inflammation are differently expressed in whole blood between patients who do and do not progress to inflammatory arthritis (IA). We studied RNA expression levels of a set of inflammatory and immune response genes that was previously compiled to identify markers of progression in other inflammatory conditions [8, 9] and now used to identify markers of progression to clinical IA in patients with arthralgia.

## Methods

### Patients

Patients were consecutively included in the Leiden CSA cohort [1]. All patients presented at the outpatient clinic with recent-onset (< 1 year) arthralgia of the small joints without clinical arthritis and were, according to the clinical expertise of the rheumatologist, suspicious for progression to RA. Baseline visits consisted of physical examination, blood sampling (including PAXgene tubes), and an MRI of hand and foot. Autoantibody status was not known at inclusion, as general practitioners were, in line with the Dutch guidelines, discouraged to determine autoantibodies. Follow-up visits were scheduled at 4, 12, and 24 months. When necessary, for instance in case of an increase of symptoms or when patients experienced joint swelling, additional visits were planned. Patients were followed until development of clinical IA, determined by the rheumatologist at physical examination. During follow-up (and before the primary outcome was reached), treatment with disease-modifying antirheumatic drugs (DMARDs) (including steroids) was not allowed. The date of censoring was the date of reviewing the medical records or an earlier date in case patients were lost to follow-up. All medical files were reviewed until July 2019.

Between April 2012 and March 2015, 255 patients were included. PAXgene tubes were not collected in 14 patients. Of the remaining 241 patients, five were included in a randomized placebo-controlled trial and were excluded here because of possible DMARD treatment leaving 236 patients (Additional Figure 1).

### Dual color reverse transcription multiplex ligation-dependent probe amplification (dcRT-MLPA)

#### RNA isolation

RNA from baseline whole blood in PAXgene tubes was extracted using PAXgene Blood RNA kits (BD Biosciences, Franklin Lakes, NJ) according to the manufacturers’ protocol. RNA yield was determined by NanoDrop ND-1000 spectrophotometer (NanoDrop Technologies, Wilmington, DE).

#### dcRT-MLPA assays

RNA expression was determined for 133 genes of the innate and adaptive immune system by dcRT-MLPA (Additional file 1) and was performed as described previously [8]. Trace data were analyzed using GeneMapper software 5 (Applied Biosystems). The areas of each assigned peak (in arbitrary units) were exported for further analysis in Microsoft Excel spreadsheet software or R-Project and log2 transformed. Results from target genes were calculated relative to the geometric average signal of selected control genes [10] (e.g., four housekeeping genes: ABR, GUSB, GAPDH, and B2M), and the percentage standard deviation was calculated to determine which control gene was most stably expressed across the evaluated samples. In our study, this was glyceraldehyde 3-phosphate dehydrogenase (GAPDH). Signals below the value for noise cutoff (log2 transformed peak area ≤ 7.64) were assigned the threshold value.

### Quantitative polymerase chain reactions (qPCRs)

RNA was converted to cDNA using GoScript™ Reverse Transcriptase Kit (A5001, Promega) as per manufacturer’s instructions. qPCR was performed with TaqMan™ Gene Expression Master Mix (4369016, Thermo Fisher) and TaqMan™ Gene Expression Assay (4331182, Thermo Fisher) of target genes (Assay IDs: IFNG Hs00989291 and IL-7R Hs00902334) and GAPDH as a control (Assay ID: Hs02786624).

### Statistical analyses

Cox proportional hazard models were used to associate time-to-event with gene expression level at inclusion, while adjusting for age, gender, and assay plate. The false discovery rate (FDR) was used to correct for multiple testing. Genes with a significantly differential expression were subsequently studied for mutual independence in their association with IA development. Resulting mutually independent genes were further investigated for their independence with respect to known risk factors that were previously found to be associated with IA development: CRP, anti-citrullinated protein antibody (ACPA), and subclinical joint inflammation [1]. In addition, genes with significantly different expression were also tested with RA as outcome. RA was defined as a clinical diagnosis plus initiation of DMARD treatment and/or fulfillment of 1987/2010 criteria at the time of IA development. We choose not to restrict to 2010 criteria positivity as ACPA-negative patients can only fulfill this definition if they have > 10 involved joints. Intercorrelation between significant genes was tested by Pearson’s correlation.

In parallel, we conducted a sensitivity analysis by omitting the measurements with values below the noise cutoff (peak area ≤ 7.64). While this analysis is more conservative, as additional samples are excluded, it does not depend on the assumptions made for imputing values measured below the noise cutoff, i.e., whether all missing values can be explained by a too low expression.

*p* values < 0.05 were considered significant. R 3.6.0 was used.

## Results

### Patient characteristics

Twenty percent of CSA patients developed IA after a median of 3.6 months (IQR 1.6–10.7) follow-up (Additional Table 1). The non-progressing patients were followed for median of 24 months (IQR 12–26).

### dcRT-MLPA shows six downregulated genes, associated with IA development

Measurement of 3 genes showed no expression, suggesting failure of the signal. The remaining 130 genes were analyzed. After correction for multiple testing and adjusting for age, gender, and assay plate, six genes were significantly associated with IA development, namely IFNG, phosphate regulating endopeptidase homolog X-linked (PHEX), insulin growth factor-1 (IGF-1), IL-7R, cluster of differentiation-19 (CD19), and C-C chemokine receptor type 7 (CCR7) (ordered by significance; FDR corrected *p* value ranges 0.019–0.037, Table 1; complete list Additional Table 2). IFNG was only weakly expressed in whole blood, and to confirm dcRT-MLPA data, expression was also measured by qPCR (Additional Figure 2A-B). Because dcRT-MLPA findings were not evidently reproduced by qPCR for IFNG (*p* = 0.068, *ρ* = −0.17), this gene was excluded from further analyses. All five remaining genes showed lower expression levels at baseline for the patients who developed IA.

**Table 1:** Associations of significant top genes at presentation with clinically suspect arthralgia and progression to IA

	Multivariable analyses^**a**^				Multivariable analyses^**b**^			Multivariable analyses^**c**^		
	Coefficient	exp (coef)	***p*** value^**^**^	***p*** value FDR	Coefficient	exp (coef)	***p*** value	Coefficient	exp (coef)	***p*** value
**IFNG**	− 0.38	0.68	< 0.001	0.019	–	–	–	–	–	–
**PHEX**	− 0.73	0.48	< 0.001	0.019	–	–	–	–	–	–
**IGF-1**	− 0.77	0.46	< 0.001	0.028	− 0.51	0.60	0.046	0.69	0.50	0.005
**IL-7R**	− 0.66	0.52	< 0.001	0.031	− 0.51	0.60	0.025	0.48	0.62	0.039
**CD19**	− 1.43	0.24	0.001	0.037	− 0.41	0.66	0.385	–	–	–
**CCR7**	− 1.33	0.26	0.002	0.037	− 0.41	0.67	0.603	–	–	–

^a^Multivariable analyses were adjusted for age, gender, and assay plate. ^^^*p* values were significant after FDR correction

^b^Multivariable analyses were adjusted for age, gender, assay plate, and genes (IGF-1, IL-7R, CD19, and CCR7; PHEX was not included because of high correlation with IGF-1; IFNG was not included because of low expression and insufficient replication of expression by qPCR)

^c^Multivariable analysis: genes that were significantly associated with IA development in analysis b were also corrected for ACPA, CRP, and subclinical joint inflammation, in addition to age, gender, assay plate

*Abbreviation*: *exp. (coef)* exponential coefficient

### IL-7R and IGF-1 independently associated with IA development

**Fig. 1:**
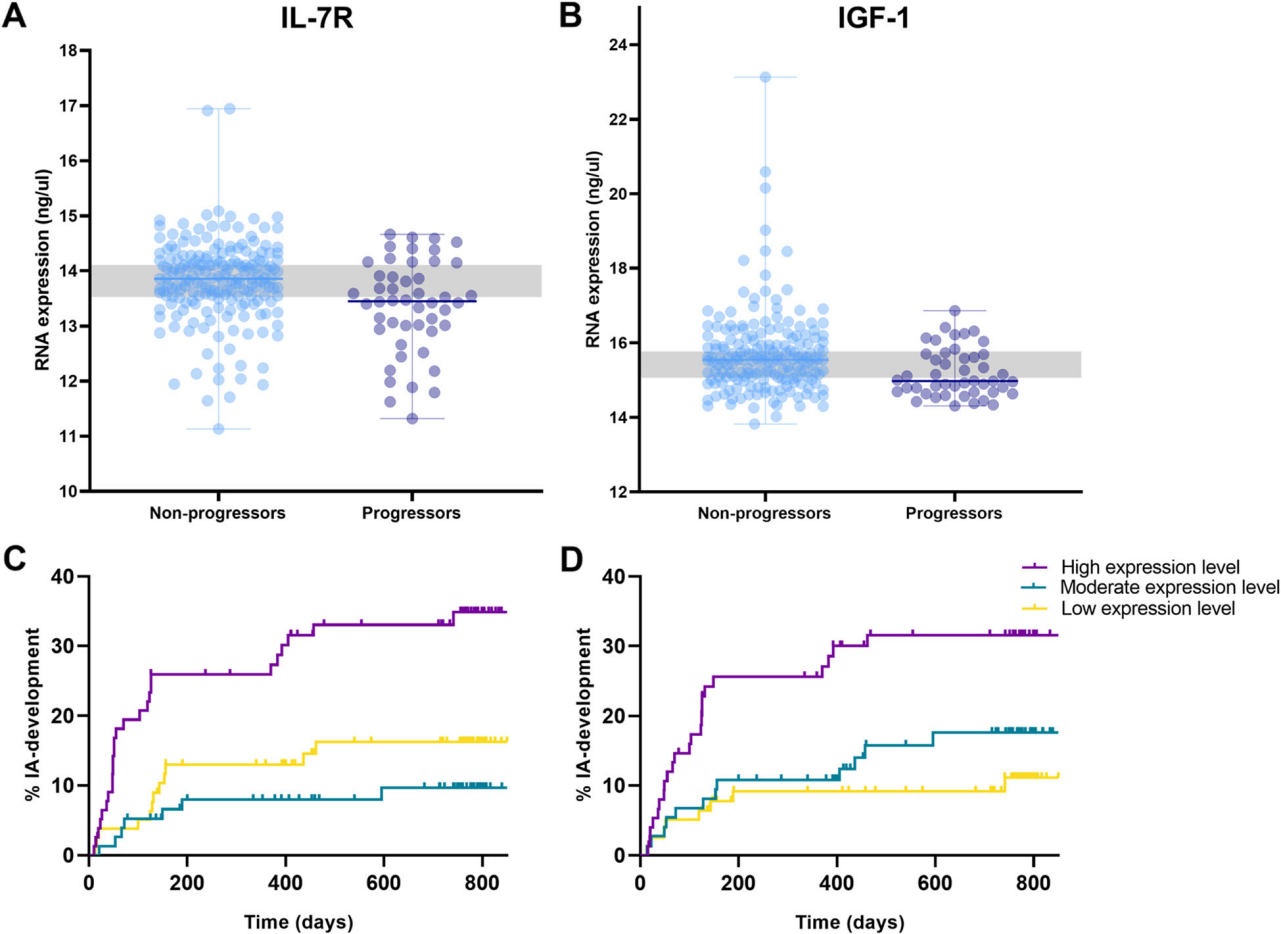
Different RNA expression between progressors and non-progressors (**a**, **b**) and association between RNA expression of IL-7R (**c**) and IGF-1 (**d**) IA development. **a**, **b** Differences of RNA expression between patients who did and did not progress to inflammatory arthritis. RNA expression levels grouped by patients that progressed to IA during follow-up (dark blue dots) and those that did not progress (light blue dots). Patients are categorized into tertiles based on the gene expression levels to create three groups of equal size; lowest expression values below the gray square (e.g., high risk), expression levels within the gray square are of moderate risk, and patients with the lowest risk are depicted above the square. **c**, **d** Association between RNA expression of IL-7R (**c**) and IGF-1 (**d**) at presentation with clinically suspect arthralgia and development of inflammatory arthritis over time. Vertical lines indicate that a patient is censored. Patients were categorized into tertiles based on the gene expression levels to create three groups of equal size; lowest expression values, and thus the highest risk for IA development, are represent in purple; moderate risk in green and lowest risk in yellow. Visual representation of the data was restricted to 850 days follow-up since thereafter the numbers of patients was small

Next, the dcRT-MLPA-expression levels of five genes were studied for mutual independence in their association with IA development. As PHEX and IGF-1 were highly correlated with each other (*ρ* = 0.97, Additional Figure 3), we only added IGF-1 to the multivariable model and not PHEX, as IGF-1 showed a slightly higher effect size and literature suggests a relation between RA and IGF-1. A multivariable model including IGF-1, IL-7R, CD19, and CCR7 revealed that expression of IL-7R and IGF-1 was independently associated with IA development (*p* = 0.025, *p* = 0.046, respectively; Table 1 and Fig. 1). Similarly, when PHEX was added instead of IGF-1, IL-7R and PHEX were independently associated with IA development (*p* = 0.024, *p* = 0.030, respectively; Additional Table 3& Figure 4), thus indicating added value in the biomarkers signature for IA development.

### IGF-1 and IL-7R were independently associated when adding clinical predictors

Subsequently, we studied whether the transcriptomic biomarkers obtained were associated with IA development independently of known risk factors (CRP, ACPA, and MRI-detected subclinical joint inflammation). Indeed, both, IL-7R and IGF-1, remained independently associated with IA development (*p* = 0.039, *p* = 0.005, respectively: Table 1). Also, when PHEX was studied instead of IGF-1, an independent association was observed (PHEX; *p* = 0.003; effect size = − 0.68).

**Fig. 2:**
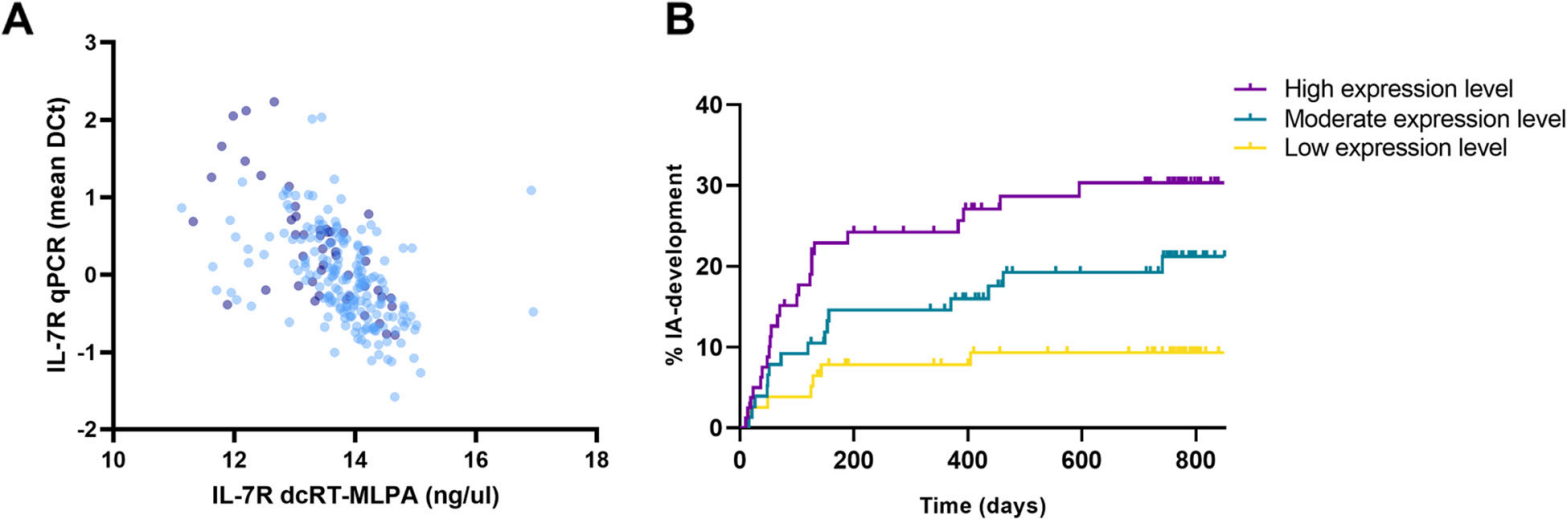
Correlation between qPCR and dcRT-MLPA IL-7R gene expression (**a**) and association between qPCR IL-7R and IA development (**b**). **a** Correlation between qPCR and dcRT-MLPA IL-7R gene expression data (*p* < 0.001, *ρ* = − 0.56). Patients that progressed to IA during follow-up (dark blue dots) and those that did not progress (light blue dots). **b** Association between qPCR IL-7R expression at presentation with clinically suspect arthralgia and development of inflammatory arthritis over time. Vertical lines indicate that a patient is censored. Patients were categorized into tertiles based on the gene expression levels to create three groups of equal size; highest expression values, and thus the highest risk for IA development, are represent in purple; moderate risk in green and lowest risk in yellow. Visual representation of the data was restricted to 850 days follow-up since thereafter the numbers of patients was small

The reproducibility of the results of IL-7R was evaluated by qPCR. Among others because of the mutual dependency, neither IGF-1 nor PHEX was tested by qPCR. The qPCR data of IL-7R correlated with dcRT-MLPA results (*p* < 0.001, *ρ* = − 0.56, Fig. 2), confirming the robustness of the transcriptomic outcome. qPCR IL-7R expression was, as expected, also associated with IA development and remained significantly associated after adjustment of known risk factors (*p* = 0.002, Fig. 2).

Finally analyses were repeated with RA development as outcome. Il-7R and IGF-1 were significantly associated, also when correcting for the other genes and for the described clinical markers (Additional Table 4).

### Sensitivity analyses

In sensitivity analysis excluding signals with peak area ≤ 7.64, 19 genes were associated with IA development (corrected for multiple testing and adjusted for age, gender, and assay plate; Additional Table 5 & Figure 5). The 5 genes (IL-7R, IGF-1, PHEX, CD19, CCR7) were among these 19 genes. Of these genes, 13 remained independently associated with IA development when added separately to known clinical predictors, including IL7-R and IGF-1 (Additional Table 6). IFNG was low expressed, as showed above, and when all signals below the value for noise cutoff were excluded, the dcRT-MLPA results strongly correlated with the qPCR data (*p* < 0.001, Additional Figure 2), confirming technical reproducibility when leaving out too low signals. However, the association with IA development was lost (*p* = 0.34; effect size = − 0.31). Thus, IFNG was expressed at low levels and in the current data not consistently associated with IA development.

## Discussion

Gene expression could be helpful in discriminating subsets of arthralgia patients who will progress to RA. We performed a longitudinal study on the RNA expression of 133 genes coding for inflammation and immunity in patients with CSA and observed that five genes were associated with IA development. From the identified genes IL-7R and IGF-1 expression levels were associated with IA and RA development independently from each other and from known RA risk factors.

We studied a comprehensive set of genes with known functions using dcRT-MLPA. The candidate genes were selected based on their known role in inflammation or immunity and their previously reported identification as markers of progression in patients with other inflammatory disorders [8]. We have not used a hypothesis-free approach such as RNA sequencing in this set of 236 patients to avoid multiple testing and subsequent false-positive results. We also applied a stringent approach when reporting mainly on IL-7R and IGF-1 in association with IA development. Thus, it is possible that we excluded other potential discriminating genes from our top five list by this approach. Notably, the sensitivity analysis showed more significant findings than the main analyses. However, at this stage, we considered false negativity a lesser concern. Towards future research, whole blood expression of IL-7R and IGF-1 genes should be validated in an independent set of arthralgia patients and genes identified in the sensitivity analysis should be further explored. Furthermore, as mentioned, a hypothesis-free approach could also identify novel markers differentiating arthralgia patients who do or do not develop IA or RA.

Previous studies have shown that, at protein level in early arthritis patients, low IL-7 levels were predictive for progression to RA, particularly in ACPA-negative disease [11]. Likewise, but at transcriptome level, in autoantibody-positive arthralgia patients, risk factors for progression to arthritis were reported to be involved in cytokine- and chemokine-mediated immunity, including low levels of IFNG and IL-7R [5]. Concomitantly, our results showing lower expression of IL-7R levels in patients who developed IA are in line with these data.

In RA, low levels of IL-7R expression on all circulating CD4+ and CD8+ T cells, NK-T cells and monocytes was found, which led to a disturbed T cell homeostasis. In addition, IL-7R is not expressed on mature B cells, but downregulation of the receptor on pre B cells has been reported in RA [12]. These IL-7R+ B cells seemed to have a proinflammatory role in arthritis, suggesting that the IL-7/IL-7R system might be a potential drug target.

IGF-1 was highly intercorrelated with PHEX and both genes showed similar findings when analyzed separately. A recent meta-analysis reported that serum levels of IGF-1 were lower in RA than in healthy controls [13], which directionality is in line with the present results in arthralgia patients. These results indicate that downregulation of IGF-1 plays a role in the pathogenesis of RA. Moreover, in RA patients, downregulation of IGF-1 expression was reported to be associated with the overexpression of tumor necrosis factor-alpha (TNF-α) and interleukin-6 (IL-6), which are two major and crucial proinflammatory cytokines in RA [14]. Also supportive of our current findings is the increase of IGF-1 after anti-TNF treatment that has been reported. Interestingly, kinase inhibitors of the IGF-1 receptor have been proposed as new specific drug-targets for RA, as these can inhibit the IGF-system signaling pathway [15].

In autoimmune diseases, expression of IGF-1 is disturbed, resulting in low levels of IGF-1 on regulatory T cells (Treg), and thus an imbalance in active suppression of inflammation and immune responses has been observed [16]. Moreover, cumulative evidence points to a role of the IGF-1/IGF-1R signaling pathway in regulating the immune response, and Treg cell proliferation by IGF-1 based therapies is proposed as a therapeutic avenue for the treatment of autoimmune and inflammatory diseases.

Thus, both IL-7R and IGF-1 genes play a significant role in T cell signaling. In addition, T cell subset dysregulation predates the onset of IA in ACPA-positive arthralgia patients, represented by reduced naïve T cells and reduced Tregs [7]. Even though these subsets are part of the CD4+ T cell pool, they are generated through different mechanisms thus reflecting a separate immunological state of the individual and contributing to the risk of progression to IA. Taken together, our data show that low expression levels of IL-7R and IGF-1 genes might influence dysregulation of T cell subsets and, thus, supports a T cell mediation origin of in the phase of arthralgia, before the progression to IA.

Previous gene expression studies in longitudinal cohorts were done in patients with autoantibody-positive arthralgia patients [4, 5]. Identification of CSA by rheumatologists in this study was independent on the presence of ACPA and consequently both ACPA-positive and ACPA-negative patients were included. Hence, we studied different candidate genes in a different population. Studying both, ACPA-positive and ACPA-negative arthralgia, is relevant as both subsets of RA might have differential pathogenesis. The current findings on IL-7R and IGF-1 were statistically independent of ACPA. The role of these genes in both subsets of RA is a subject for further research.

In our view, the present results increase the comprehension of processes mediating progression from arthralgia to clinical arthritis and RA. In the long run, IL-7R and IGF-1 can become prognostically helpful. However, this would require more work, such as external validation in other arthralgia cohorts and determination of a cutoff. The latter should preferably not be done on arthralgia patients only but also includes data from healthy controls. Thus, while encouraging that both markers were associated with IA development independent of regularly used markers, more efforts are needed to determine the clinical utility for daily practice.

## Conclusions

In conclusion, several immunity-related genes were differently expressed in arthralgia patients who progressed to RA. Of these, the expression of IL-7R and IGF-1 were independent of known clinical predictors and could become helpful in prognostication of CSA patients. Furthermore, the present data may support the previously published notion that T cell characteristics mediate progression from arthralgia to clinical arthritis [7].

## Supplementary Information

**Additional file 1 Supplementary file 1.** Detailed description of methods.

## Data Availability

The datasets used and/or analyzed during the current study are available from the corresponding author on reasonable request.

## Abbreviations

ACPA: Anti-citrullinated protein antibody
CCR7: C-C chemokine receptor type 7
CD19: Cluster of differentiation-19
CRP: C-reactive protein
CSA: Clinically suspect arthralgia
dcRT-MLPA: Dual-color reverse transcription multiplex ligation-dependent probe amplification
DMARD: Disease-modifying antirheumatic drugs
FDR: False discovery rate
GAPDH: Glyceraldehyde 3-phosphate dehydrogenase
IA: Inflammatory arthritis
IFNG: Interferon gamma
IGF-1: Insulin growth factor-1
IL-7R: Interleukin-7 receptor
PHEX: Phosphate regulating endopeptidase homolog X-linked
qPCR: Quantitative polymerase chain reactions
RA: Rheumatoid arthritis
TNF-α: Tumor necrosis factor-alpha
Treg: Regulatory T cells
