# Correction to: Gene expression identifies patients who develop inflammatory arthritis in a clinically suspect arthralgia cohort

**DOI:** 10.1186/s13075-020-02402-w

**Published:** 2020-12-20

**Authors:** Ellis Niemantsverdriet, Erik B. van den Akker, Debbie M. Boeters, Susan J. F. van den Eeden, Annemieke Geluk, Annette H. M. van der Helm-van Mil

**Affiliations:** 1 Department of Rheumatology, Leiden University Medical Center, PO Box 9600, Leiden, 2300 RC The Netherlands; 2 Department of Biomedical Data Sciences, Leiden University Medical Center, Leiden, The Netherlands; 3 Pattern Recognition & Bioinformatics, Delft University of Technology, Delft, The Netherlands; 4 Department of Infectious Diseases/Immunohematology and Blood Transfusion, Leiden University Medical Center, Leiden, The Netherlands; 5 Department of Rheumatology, Erasmus Medical Center, Rotterdam, The Netherlands

**Correction to: Arthritis Res Ther (2020) 22:266**

**https://doi.org/10.1186/s13075-020-02361-2**

Following publication of the original article [1], a typesetting error was reported. The equal contribution note for this article was incorrect. The corrected statement is given below.

† Ellis Niemantsverdriet and Erik B. van den Akker contributed equally and are joint first authors.

† Annemieke Geluk and Annette H. M. van der Helm-van Mil contributed equally and are joint last authors.

The original article [1] has been corrected.
