# Supplementary material for: Gene expression identifies patients who develop inflammatory arthritis in a clinically suspect arthralgia cohort
Source: Arthritis Res Ther. 2020 Nov 9;22:266. doi: 10.1186/s13075-020-02361-2 (PMC7653888; doi:10.1186/s13075-020-02361-2)
Supplement: Supplementary file 1 — Additional file 1 Supplementary file 1. Detailed description of methods. [file 13075_2020_2361_MOESM1_ESM.docx]

# **Supplementary file 1.** Detailed description of methods.

*Dual color Reverse-Transcription Multiplex Ligation-dependent Probe Amplification (dcRT-MLPA) assay* For each target-specific sequence, a specific RT primer was designed located immediately downstream of the half-probes target sequences (Sigma-Aldrich, Saint Louis, MO). RNA samples (2.5 μl of a 50 ng/μl solution) were reverse transcribed with 1x MMLV reverse transcriptase buffer, dNTPs (0.4 mM of each nucleotide), and 80 nM of the target specific RT primers in a final volume of 4.5 μl. After heating for 1 min to 80°C and incubation for 5 min at 45°C, 30U MMLV reverse transcriptase (Promega, Madison, WI) was added and incubated for 15 min at 37°C before heat inactivation of the enzyme for 2 min at 98°C. Subsequently, half-probes (6 nM) were added to the reaction, heat denatured for 1 min at 95°C followed by hybridization for 16 h at 60°C. Ligation of the annealed half-probes was performed for 15 min at 54°C by ligase-65 followed by heat inactivation for 5 min at 98°C. Ligation products were amplified by PCR. Thermal cycling conditions encompassed: 33 cycles of 30s/95°C, 30s/58°C, and 60s/72°C, followed by 1 cycle of 20min/72°C. PCR products were diluted 1:10 in HiDi formamide containing 400 HD ROX size standard and analyzed on an Applied Biosystems 3730 capillary sequencer in GeneScan mode (Applied Biosystems, Foster City, CA). MLPA reagents were from MRC-Holland (Amsterdam, The Netherlands).

**Supplementary Figure 1.** Flowchart of included CSA-patients.

CSA-patients included between
April-2012 and March-2015
(n=255)

CSA-patients with RNA isolation and in which MLPA was performed (n=241)

Patients without PAXgene sampling (n=14)

CSA-patients included in the study (n=236)

Patients included in placebo-controlled randomized clinical trial (n=5)

**Supplementary Figure 2.** Differences of IFNG RNA expression between patients who did and did not progress to inflammatory arthritis (A), correlation between qPCR- and dcRT-MLPA IFNG gene expression data (B), and association between RNA expression of IFGN (C,D with and without 7.64 cut-off values, respectively) at presentation with clinically suspect arthralgia and development of inflammatory arthritis over time.
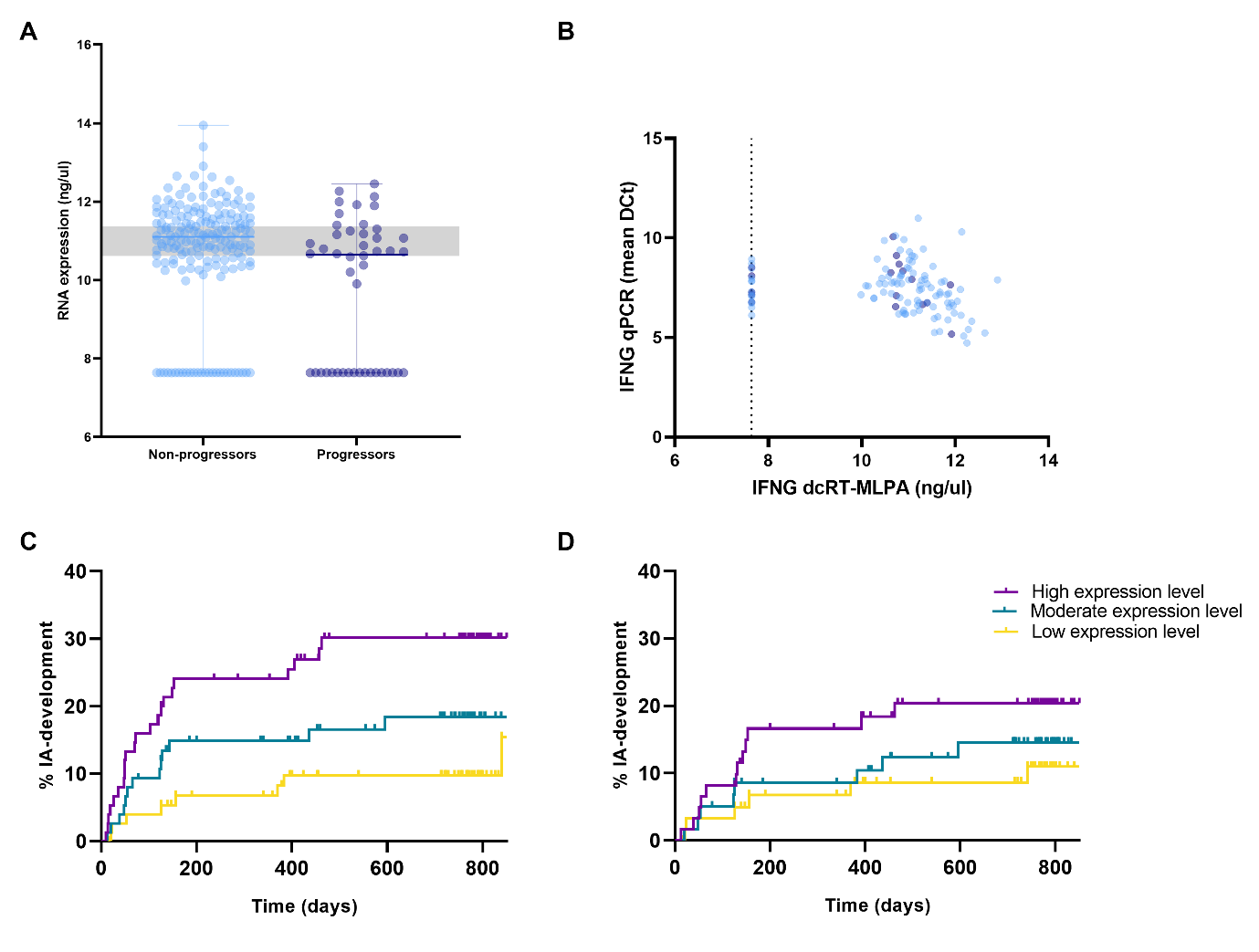
A: RNA expression levels grouped by patients that progressed to IA during follow-up (dark blue dots) and those that did not progress (light blue dots). Patients are categorized into tertiles based on the gene expression levels to create three groups of equal size; lowest expression values below the grey square (e.g. high risk), expression levels within the grey square are of moderate risk, and patients with the lowest risk are depicted above the square.
B: Correlation between qPCR and dcRT-MLPA IFNG gene expression data (p=0.068, ρ=-0.17). When signals below the value for noise cut-off (peak area ≤7.64, n=54, dotted line) were excluded from analysis, correlation between to techniques was significant (p<0.001, ρ=-0.41). Patients that progressed to IA during follow-up (dark blue dots) and those that did not progress (light blue dots).
C,D: Association with IA-development was found in case all signals were included (C, p<0.001; effect size -0.38) but not when signals below the value for noise cut-off (peak area ≤7.64) were excluded (D, p=0.34; effect size -0.31). Hence the association between INFG and IA-development was not consistent. Vertical lines indicate that a patient is censored. Patients were categorized into tertiles based on the gene expression levels to create three groups of equal size; lowest expression values, and thus the highest risk for IA-development, are represent in purple; moderate risk in green and lowest risk in yellow. Visual representation of the data was restricted to 850 days follow-up since thereafter the numbers of patients was small.

**Supplementary Figure 3.** Intercorrelation of the six significant top hit genes.


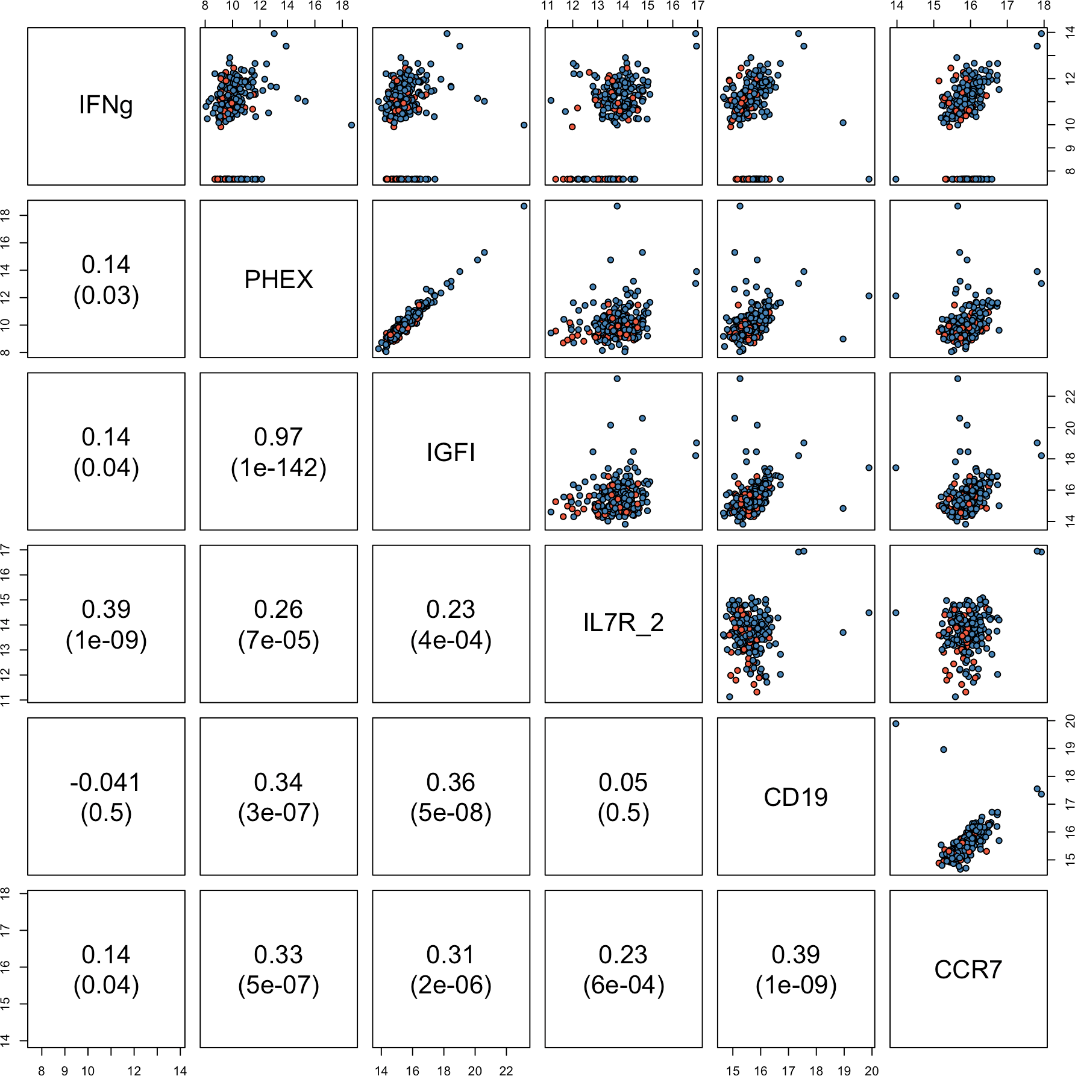


**Supplementary Figure 4.** Differences of PHEX RNA expression between patients who did and did not progress to inflammatory arthritis (A) and association between RNA expression of PHEX (B) at presentation with clinically suspect arthralgia and development of inflammatory arthritis over time.

**
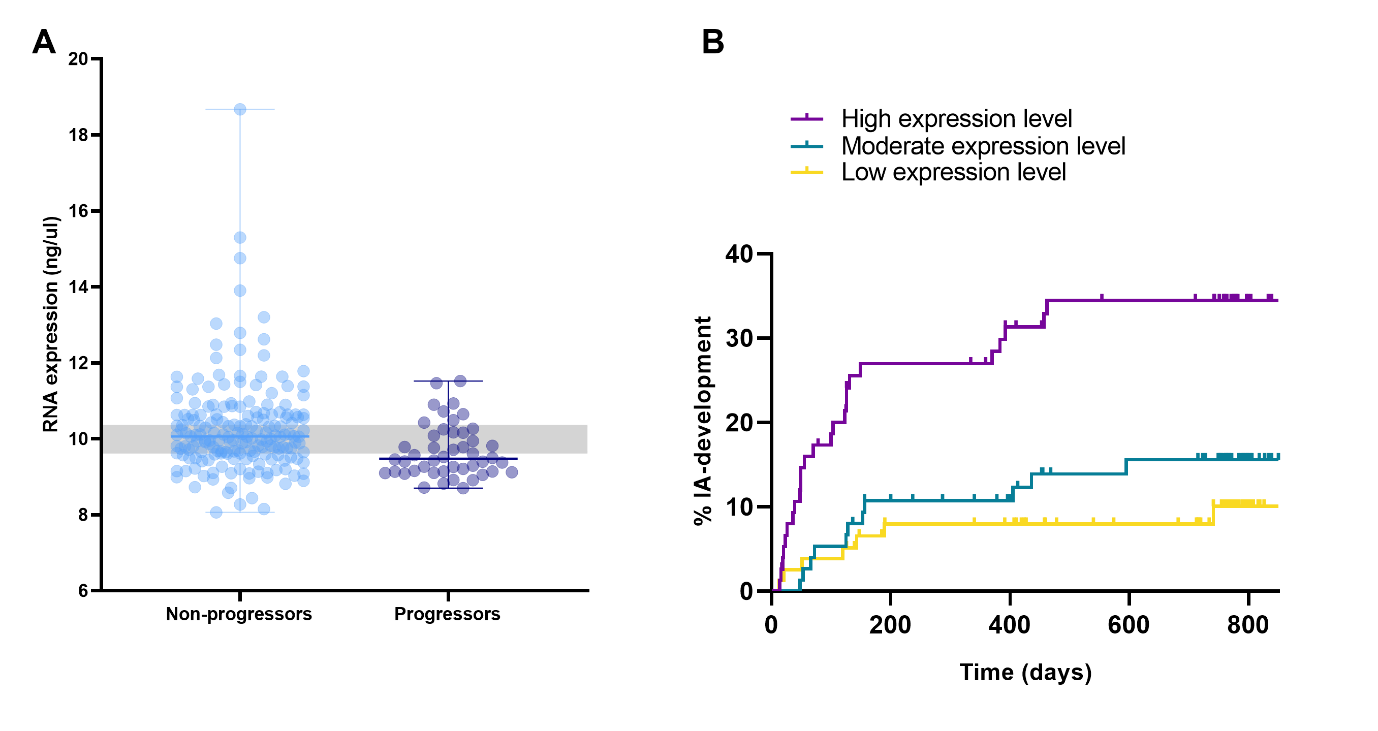
**

A: RNA expression levels grouped by patients that progressed to IA during follow-up (dark blue dots) and those that did not progress (light blue dots). Patients are categorized into tertiles based on the gene expression levels to create three groups of equal size; lowest expression values below the grey square (e.g. high risk), expression levels within the grey square are of moderate risk, and patients with the lowest risk are depicted above the square.

B: Vertical lines indicate that a patient is censored. Patients were categorized into tertiles based on the gene expression levels to create three groups of equal size; lowest expression values, and thus the highest risk for IA-development, are represent in purple; moderate risk in green and lowest risk in yellow. Visual representation of the data was restricted to 850 days follow-up since thereafter the numbers of patients was small.

**Supplementary Figure 5.** Sensitivity analysis by omitting measurements with peak area ≤7.64 - Heatmap of the association between RNA expression at presentation with clinically suspect arthralgia and development of inflammatory arthritis over time.


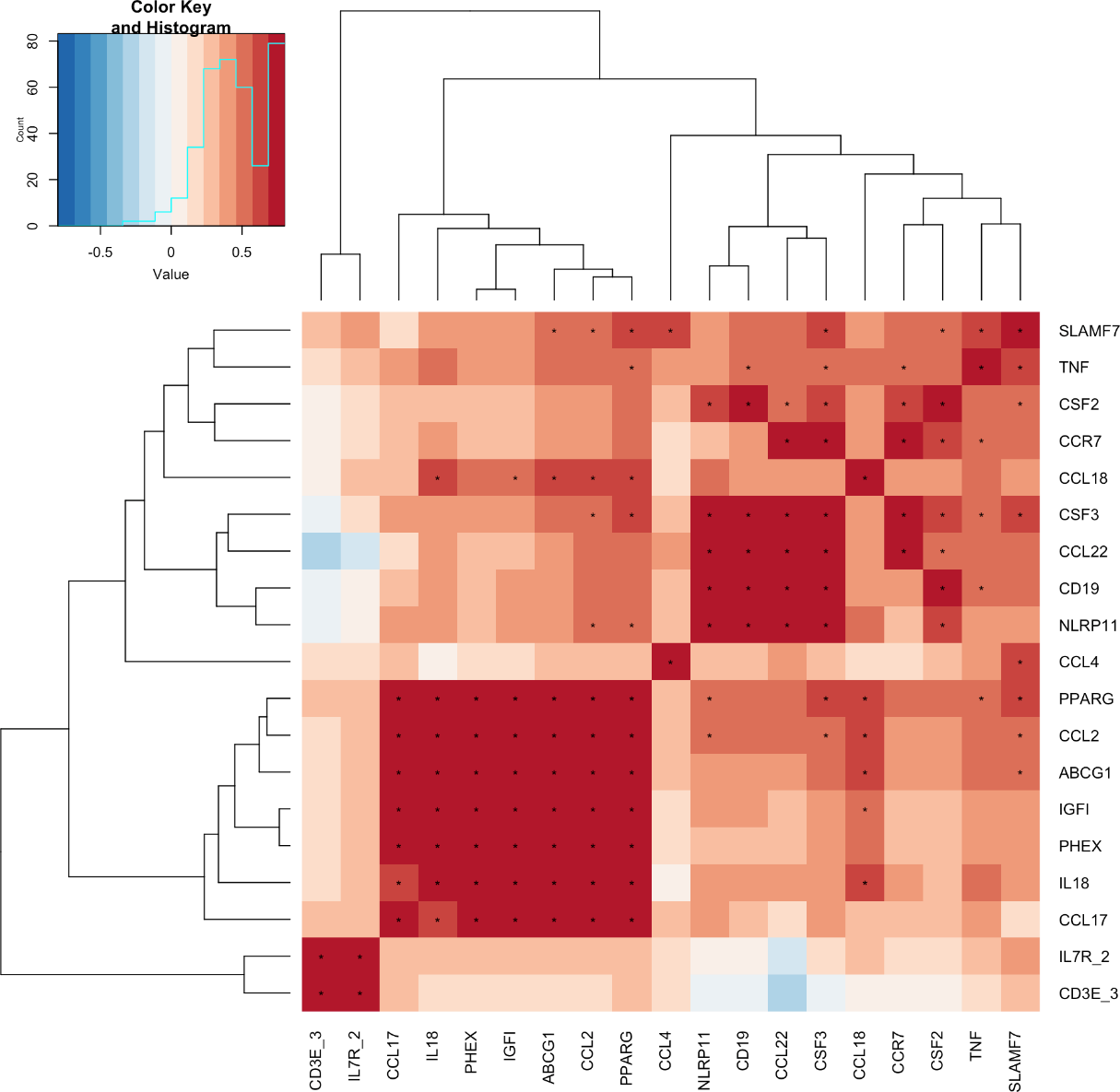


SLAMF7

TNF

CSF2

CCR7

CCL18

CSF3

CCL22

CD19

NLRP11

CCL4

PPARG

CCL2

ABCG1

IGF-1

PHEX

IL18

CCL17

IL-7R

CD3E_3

CCL22

CSF3

CCL18

CCR7

CSF2

TNF

SLAMF7

CD3E_3

IL-7R

CCL17

IL18

PHEX

IGF-1

ABCG1

CCL2

PPARG

CCL4

NLRP11

CD19

Heatmap of 19 genes that were significantly differentially expressed between 236 arthralgia-patients (false discovery rate <0.05). Red color indicates positive correlations between genes, blue represent negative correlations between genes, and white indicates no associations between genes.

**Supplementary Table 1.** Baseline characteristics of CSA-patients.

|  | **Total population**  **(n=236)** | **Patients that progressed to IA**  **(n=48)** | **Non-progressing patients**  **(n=188)** | **p-value** |
| --- | --- | --- | --- | --- |
| Age, mean (SD) | 43.9 (13.1) | 45.0 (13.4) | 43.6 (13.0) | 0.383 |
| Female, n (%) | 183 (78) | 37 (77) | 146 (78) | 0.932 |
| Sympt dur (wks), median (IQR) | 17 (9-32) | 17.5 (9-30) | 17 (9-33) | 0.742 |
| 68-TJC, median (IQR) | 5 (3-10) | 5 (4-9) | 6 (2-10) | 0.966 |
| CRP mg/L, median (IQR) | 3 (3-4.6) | 3 (3-11.6) | 3 (3-4) | 0.016 |
| RF positive, n (%) | 48 (20) | 25 (52) | 23 (12) | <0.001 |
| ACPA positive, n (%) | 31 (13) | 22 (46) | 9 (5) | <0.001 |

At baseline CRP, percentage of ACPA-positive and RF-positive were significantly different between patients that did and did not progressed to IA, with more severe serology measures in progressors. Statistical analyses were performed with Mann-Whitney U tests and Person Chi-Square tests.

From the 22 patients that progressed to IA, 19 patients had RA at the time of IA-development.

ACPA, anti-citrullinated protein antibodies; CRP, C-reactive protein; IQR, inter quartile range; RF, rheumatoid factor; SD standard deviation; Sympt dur, symptom duration; TJC, tender joint count; wks, weeks.

**Supplementary Table 2.** Complete overview of all candidate gene-expression biomarkers at presentation with clinically suspect arthralgia and the association between expression at baseline and inflammatory arthritis development – identifying 6 significantly expressed top hit genes with FDR <0.05.

|  | **coefficient** | **exp(coef)** | **SE(coef)** | **p-value** | **p-value FDR** |
| --- | --- | --- | --- | --- | --- |
| **IFNG** | -0.38 | 0.68 | 0.10 | <0.001 | 0.019 |
| **PHEX** | -0.73 | 0.48 | 0.20 | <0.001 | 0.019 |
| **IGF-I** | -0.77 | 0.46 | 0.23 | 0.001 | 0.028 |
| **IL-7R** | -0.66 | 0.52 | 0.20 | 0.001 | 0.031 |
| **CD19** | -1.43 | 0.24 | 0.45 | 0.001 | 0.037 |
| **CCR7** | -1.33 | 0.26 | 0.42 | 0.002 | 0.037 |
| **PARK2v123** | -0.29 | 0.75 | 0.10 | 0.005 | 0.095 |
| **SLAMF7** | -0.86 | 0.42 | 0.32 | 0.006 | 0.104 |
| **TLR10** | -0.94 | 0.39 | 0.36 | 0.008 | 0.115 |
| **EGF** | -0.54 | 0.58 | 0.21 | 0.009 | 0.115 |
| **CSF3** | -0.53 | 0.59 | 0.21 | 0.012 | 0.140 |
| **NLRP11** | -0.60 | 0.55 | 0.24 | 0.013 | 0.140 |
| **TGFB1** | 1.14 | 3.12 | 0.48 | 0.017 | 0.157 |
| **GBP1** | 0.32 | 1.38 | 0.14 | 0.019 | 0.157 |
| **BMP6** | -0.56 | 0.57 | 0.24 | 0.019 | 0.157 |
| **TNFRSF18** | -0.97 | 0.38 | 0.41 | 0.019 | 0.157 |
| **NEDD4L** | -1.06 | 0.35 | 0.46 | 0.022 | 0.167 |
| **CD3E** | -0.28 | 0.75 | 0.13 | 0.025 | 0.182 |
| **IL8** | -0.24 | 0.79 | 0.11 | 0.028 | 0.191 |
| **IFIT2** | 0.32 | 1.38 | 0.15 | 0.033 | 0.208 |
| **LYN** | 0.78 | 2.18 | 0.37 | 0.034 | 0.208 |
| **IL13** | -0.35 | 0.71 | 0.17 | 0.040 | 0.228 |
| **TLR7** | -0.77 | 0.46 | 0.38 | 0.041 | 0.228 |
| **IL15** | -0.17 | 0.84 | 0.08 | 0.042 | 0.228 |
| **SOCS1** | -0.47 | 0.62 | 0.24 | 0.051 | 0.248 |
| **CCL4** | -0.34 | 0.71 | 0.18 | 0.051 | 0.248 |
| **TNF** | -0.29 | 0.75 | 0.15 | 0.051 | 0.248 |
| **MMP2** | -0.26 | 0.77 | 0.14 | 0.054 | 0.249 |
| **IL12A** | -0.43 | 0.65 | 0.23 | 0.056 | 0.249 |
| **CD14** | 0.77 | 2.15 | 0.41 | 0.058 | 0.252 |
| **CCL2** | -0.13 | 0.88 | 0.07 | 0.060 | 0.252 |
| **CD46** | 0.71 | 2.04 | 0.38 | 0.064 | 0.257 |
| **IFIT5** | -0.25 | 0.78 | 0.14 | 0.065 | 0.257 |
| **CD68** | 0.46 | 1.58 | 0.25 | 0.070 | 0.263 |
| **BLR1** | -0.13 | 0.88 | 0.07 | 0.071 | 0.263 |
| **IFITM3** | 0.18 | 1.20 | 0.10 | 0.076 | 0.268 |
| **TLR2** | -0.39 | 0.68 | 0.22 | 0.076 | 0.268 |
| **CSF2** | -0.36 | 0.70 | 0.20 | 0.078 | 0.268 |
| **IL18** | -0.14 | 0.87 | 0.08 | 0.083 | 0.275 |
| **IL6_3** | -0.39 | 0.68 | 0.23 | 0.085 | 0.275 |
| **CCL3** | -0.18 | 0.83 | 0.11 | 0.096 | 0.294 |
| **B2M** | -0.26 | 0.77 | 0.16 | 0.097 | 0.294 |
| **CD274** | -0.34 | 0.71 | 0.21 | 0.099 | 0.294 |
| **IL13_2** | -0.36 | 0.70 | 0.22 | 0.100 | 0.294 |
| **TOLLIP** | -0.17 | 0.84 | 0.11 | 0.103 | 0.294 |
| **FCGR1A** | 0.27 | 1.31 | 0.17 | 0.106 | 0.294 |
| **HDAC1** | 0.10 | 1.10 | 0.06 | 0.106 | 0.294 |
| **DSE** | -0.35 | 0.70 | 0.22 | 0.113 | 0.305 |
| **NOD2** | -0.29 | 0.75 | 0.18 | 0.117 | 0.310 |
| **ABR_7** | -0.20 | 0.82 | 0.13 | 0.120 | 0.313 |
| **GATA3** | -0.31 | 0.74 | 0.20 | 0.132 | 0.328 |
| **CXCL9** | -0.21 | 0.81 | 0.14 | 0.133 | 0.328 |
| **ABCG1** | -0.11 | 0.89 | 0.07 | 0.134 | 0.328 |
| **GUSB_2** | -0.55 | 0.58 | 0.39 | 0.156 | 0.363 |
| **CXCL3L1** | -0.61 | 0.54 | 0.43 | 0.158 | 0.363 |
| **MBP** | -0.27 | 0.76 | 0.19 | 0.158 | 0.363 |
| **IL2** | -0.21 | 0.81 | 0.15 | 0.159 | 0.363 |
| **IFIT3** | 0.22 | 1.25 | 0.16 | 0.166 | 0.372 |
| **FCGR1B** | 0.17 | 1.19 | 0.13 | 0.181 | 0.399 |
| **OAS1** | 0.20 | 1.22 | 0.15 | 0.189 | 0.409 |
| **PPARG** | -0.12 | 0.88 | 0.10 | 0.194 | 0.414 |
| **AIRE** | -0.22 | 0.80 | 0.17 | 0.201 | 0.421 |
| **CFH** | 0.09 | 1.10 | 0.08 | 0.211 | 0.435 |
| **GBP5** | 0.24 | 1.27 | 0.20 | 0.218 | 0.441 |
| **GUSB_1** | -0.50 | 0.60 | 0.41 | 0.220 | 0.441 |
| **NPC1** | -0.13 | 0.88 | 0.11 | 0.241 | 0.474 |
| **NLRP2** | -0.06 | 0.94 | 0.05 | 0.244 | 0.474 |
| **GBP2** | 0.26 | 1.30 | 0.24 | 0.267 | 0.511 |
| **NLRP7** | -0.22 | 0.81 | 0.20 | 0.281 | 0.529 |
| **MMP12** | -0.15 | 0.86 | 0.15 | 0.293 | 0.537 |
| **CCR6** | -0.14 | 0.87 | 0.14 | 0.293 | 0.537 |
| **IL22RA1** | -0.24 | 0.78 | 0.24 | 0.302 | 0.545 |
| **IFIH1** | 0.18 | 1.19 | 0.18 | 0.330 | 0.583 |
| **CXCL10** | -0.12 | 0.89 | 0.12 | 0.332 | 0.583 |
| **TLR6** | 0.20 | 1.22 | 0.21 | 0.345 | 0.598 |
| **IFI44L** | 0.08 | 1.08 | 0.09 | 0.366 | 0.626 |
| **CXCL7** | 0.18 | 1.19 | 0.20 | 0.373 | 0.627 |
| **CD163** | -0.37 | 0.69 | 0.42 | 0.380 | 0.627 |
| **TLR1** | 0.18 | 1.20 | 0.21 | 0.381 | 0.627 |
| **CCL17** | -0.03 | 0.97 | 0.03 | 0.397 | 0.646 |
| **IL1Ra** | 0.12 | 1.12 | 0.15 | 0.427 | 0.685 |
| **KIF1B** | 0.22 | 1.25 | 0.29 | 0.447 | 0.697 |
| **HCK** | 0.27 | 1.31 | 0.35 | 0.448 | 0.697 |
| **IL12p40** | -0.94 | 0.39 | 1254.00 | 0.453 | 0.697 |
| **CCL11** | -0.08 | 0.93 | 0.10 | 0.455 | 0.697 |
| **ABCA1** | 0.07 | 1.07 | 0.10 | 0.476 | 0.712 |
| **VEGF** | -12833 | <0.01 | 18273 | 0.483 | 0.712 |
| **TLR9** | -0.10 | 0.91 | 0.14 | 0.484 | 0.712 |
| **TAP2** | -0.13 | 0.88 | 0.19 | 0.493 | 0.712 |
| **TWIST** | -0.10 | 0.90 | 0.15 | 0.493 | 0.712 |
| **CD209** | -0.10 | 0.90 | 0.17 | 0.545 | 0.761 |
| **s100A12** | -0.04 | 0.97 | 0.06 | 0.552 | 0.761 |
| **ABR_4** | -0.14 | 0.87 | 0.23 | 0.553 | 0.761 |
| **IL6** | -0.13 | 0.87 | 0.23 | 0.553 | 0.761 |
| **HPRT** | 0.07 | 1.08 | 0.12 | 0.556 | 0.761 |
| **MSR1** | -0.06 | 0.94 | 0.10 | 0.569 | 0.771 |
| **IFI35** | -0.12 | 0.88 | 0.23 | 0.594 | 0.796 |
| **IRF7** | -0.27 | 0.76 | 0.55 | 0.618 | 0.819 |
| **IL9** | -0.05 | 0.95 | 0.12 | 0.646 | 0.830 |
| **CCL22** | -0.06 | 0.94 | 0.13 | 0.647 | 0.830 |
| **IFI16** | -0.12 | 0.89 | 0.26 | 0.647 | 0.830 |
| **ASAP1** | -0.15 | 0.86 | 0.35 | 0.658 | 0.830 |
| **PACGRv1** | 0.03 | 1.03 | 0.06 | 0.661 | 0.830 |
| **LIPE** | 0.08 | 1.09 | 0.19 | 0.664 | 0.830 |
| **TAP1** | 0.09 | 1.09 | 0.22 | 0.678 | 0.834 |
| **TNIP1** | 0.11 | 1.12 | 0.27 | 0.680 | 0.834 |
| **STAT1** | 0.08 | 1.08 | 0.19 | 0.687 | 0.835 |
| **IFI6** | 0.06 | 1.07 | 0.17 | 0.715 | 0.854 |
| **PTPRCv1** | -0.09 | 0.91 | 0.26 | 0.718 | 0.854 |
| **IL23A** | 0.10 | 1.11 | 0.29 | 0.722 | 0.854 |
| **GZMB** | -0.10 | 0.90 | 0.31 | 0.738 | 0.864 |
| **LTA4H** | -0.02 | 0.98 | 0.06 | 0.763 | 0.873 |
| **IL1A** | 0.02 | 1.02 | 0.06 | 0.769 | 0.873 |
| **CTLA4** | -0.05 | 0.95 | 0.18 | 0.771 | 0.873 |
| **CXCL4** | 0.05 | 1.05 | 0.18 | 0.772 | 0.873 |
| **NR1H3** | -0.02 | 0.98 | 0.07 | 0.784 | 0.879 |
| **GNLY** | -0.05 | 0.95 | 0.20 | 0.799 | 0.887 |
| **GZMA** | 0.03 | 1.03 | 0.14 | 0.816 | 0.899 |
| **IFI44** | 0.03 | 1.03 | 0.15 | 0.823 | 0.899 |
| **PTPRCv2** | 0.03 | 1.03 | 0.14 | 0.834 | 0.901 |
| **MARCO** | 0.02 | 1.02 | 0.10 | 0.838 | 0.901 |
| **CCL18** | 0.01 | 1.01 | 0.06 | 0.871 | 0.928 |
| **IL10** | -0.01 | 0.99 | 0.10 | 0.888 | 0.939 |
| **NLRP12** | -0.04 | 0.96 | 0.31 | 0.907 | 0.951 |
| **IDO1** | -0.03 | 0.97 | 0.29 | 0.929 | 0.966 |
| **FOXP3** | -37.09 | <0.01 | 686 | 0.957 | 0.987 |
| **OAS2** | <0.01 | 1.00 | 0.16 | 0.980 | 0.994 |
| **IL1B** | 0.01 | 1.01 | 0.27 | 0.981 | 0.994 |
| **CD36** | <0.01 | 1.00 | 0.25 | 0.989 | 0.994 |
| **CD4** | <0.01 | 1.00 | 0.26 | 0.994 | 0.994 |

Abbreviations: exp(coef), exponential coefficient; FDR, false discovery rate; SE(coef), standard error coefficient.

**Supplementary Table 3.** Multivariable analysis adjusted for age, gender, assay plate, and genes (PHEX, IL-7R, CD19, and CCR7), when PHEX was used instead of IGF-1, and the association of the genes and the chance of developing inflammatory arthritis.

|  | **coefficient** | **exp(coef)** | **p-value** |
| --- | --- | --- | --- |
| PHEX | -0.50 | 0.61 | 0.030 |
| IL-7R | -0.51 | 0.60 | 0.024 |
| CD19 | -0.43 | 0.65 | 0.358 |
| CCR7 | -0.35 | 0.71 | 0.660 |

Abbreviation: exp(coef), exponential coefficient

**Supplementary Table 4.** Multivariable analysis of the 5 significant top hit genes, adjusted for age, gender, and assay plate, and the association with RA-development.

|  | **Multivariable analyses^a^** | | | | **Multivariable analyses^b^** | | | **Multivariable analyses^c^** | | |  |
| --- | --- | --- | --- | --- | --- | --- | --- | --- | --- | --- | --- |
|  | **coefficient** | **exp(coef)** | **p-value^^^** | **p-value FDR** | **coefficient** | **exp(coef)** | **p-value** | **coefficient** | **exp(coef)** | **p-value** |  |
| **PHEX** | -0.84 | 0.43 | <0.001 | <0.001 | -- | -- | -- | -- | -- | -- |  |
| **IGF-1** | -0.91 | 0.40 | <0.001 | <0.001 | -0.51 | 0.60 | 0.046 | -0.69 | 0.50 | 0.005 |  |
| **CD19** | -1.48 | 0.23 | 0.0011 | 0.0017 | -0.41 | 0.66 | 0.385 | -- | -- | -- |  |
| **CCR7** | -1.36 | 0.26 | 0.0015 | 0.0017 | -0.41 | 0.67 | 0.603 | -- | -- | -- |  |
| **IL-7R** | -0.64 | 0.53 | 0.0017 | 0.0017 | -0.51 | 0.60 | 0.025 | -0.48 | 0.62 | 0.039 |  |

^a^Multivariable analyses were adjusted for age, gender, and assay plate. IFNG was not included because of low expression and insufficient replication of expression by qPCR. ^^^P-values were significant after FDR correction.
 ^b^Multivariable analyses were adjusted for age, gender, assay plate, and genes (IGF-1, IL-7R, CD19, and CCR7; PHEX was not included because of high correlation with IGF-1).
^C^Multivariable analysis: genes that were significantly associated with RA-development in analysis b, were also corrected for ACPA, CRP and subclinical joint inflammation, in addition to age, gender, assay plate.

Abbreviation: exp(coef), exponential coefficient.

**Supplementary Table 5.** Sensitivity analysis by omitting measurements with peak area ≤7.64 - Significant gene expression biomarkers of cox proportional hazard models, adjusted for age, gender, and assay plate, and corrected for multiple testing (FDR <0.05).

|  | **coefficient** | **exp(coef)** | **p-value** | **p-value FDR** |
| --- | --- | --- | --- | --- |
| **PHEX** | -0.73 | 0.48 | <0.001 | 0.017 |
| **CCL2** | -0.83 | 0.44 | <0.001 | 0.017 |
| **PPARG** | -0.83 | 0.43 | <0.001 | 0.017 |
| **IGFI** | -0.77 | 0.46 | 0.001 | 0.017 |
| **TNF** | -1.64 | 0.19 | 0.001 | 0.017 |
| **IL7-R** | -0.66 | 0.52 | 0.001 | 0.017 |
| **CCL4** | -1.11 | 0.33 | 0.001 | 0.017 |
| **CSF3** | -1.48 | 0.23 | 0.001 | 0.017 |
| **CCL17** | -0.60 | 0.55 | 0.001 | 0.017 |
| **CD19** | -1.43 | 0.24 | 0.001 | 0.017 |
| **ABCG1** | -0.61 | 0.54 | 0.001 | 0.017 |
| **CCL18** | -0.60 | 0.55 | 0.002 | 0.017 |
| **CCR7** | -1.33 | 0.26 | 0.002 | 0.017 |
| **CCL22** | -1.43 | 0.24 | 0.002 | 0.017 |
| **CD3E_3** | -0.63 | 0.53 | 0.002 | 0.020 |
| **NLRP11** | -1.02 | 0.36 | 0.004 | 0.029 |
| **IL18** | -0.36 | 0.70 | 0.004 | 0.032 |
| **SLAMF7** | -0.86 | 0.42 | 0.006 | 0.045 |
| **CSF2** | -0.87 | 0.42 | 0.007 | 0.045 |

Abbreviations: exp(coef), exponential coefficient; FDR, false discovery rate.

**Supplementary Table 6.** Sensitivity analysis by omitting measurements with peak area ≤7.64 - Significant gene expression biomarkers of cox proportional hazard models, when also correcting for known clinical predictors.

|  | **coefficient** | **exp(coef)** | **p-value** |
| --- | --- | --- | --- |
| **PHEX** | -0.68 | 0.51 | 0.003 |
| **IGF-1** | -0.69 | 0.50 | 0.005 |
| **IL18** | -0.39 | 0.68 | 0.007 |
| **CCL2** | -0.67 | 0.51 | 0.009 |
| **PPARG** | -0.67 | 0.51 | 0.011 |
| **CCL18** | -0.56 | 0.57 | 0.011 |
| **CCR7** | -1.30 | 0.27 | 0.012 |
| **TNF** | -1.33 | 0.27 | 0.021 |
| **CCL4** | -0.93 | 0.39 | 0.024 |
| **SLAMF7** | -0.86 | 0.42 | 0.025 |
| **IL-7R** | -0.48 | 0.62 | 0.039 |
| **CSF2** | -0.70 | 0.49 | 0.045 |
| **NLRP11** | -0.85 | 0.43 | 0.048 |
| **CD19** | -1.04 | 0.35 | 0.051 |
| **CCL22** | -0.90 | 0.41 | 0.099 |
| **CD3E_3** | -0.36 | 0.70 | 0.111 |
| **CSF3** | -0.71 | 0.49 | 0.167 |
| **ABCG1** | -0.24 | 0.79 | 0.196 |
| **CCL17** | -0.28 | 0.75 | 0.412 |

Abbreviations: exp(coef), exponential coefficient; FDR, false discovery rate.
